# Local Cooling as a Step of Treatment for Tissue Ischemia Caused by Hyaluronic Acid Injection-induced Embolism—A Report of 9 Cases

**DOI:** 10.1097/GOX.0000000000001824

**Published:** 2018-08-08

**Authors:** Chen Zhang, Chunying Ge, Chunxiao Du, Jieqi Li

**Affiliations:** From the * Institute of Plastic Surgery, Dalian University, Dalian, China; † NY Fashion Clinic, Dalian, China; ‡ Shenyang Mylike Aesthetic Hospital, Shenyang, China.

## Abstract

Hyaluronic acid injection is 1 of the most popular procedures in facial rejuvenation and augmentation. It is widely popular in the cosmetic surgery due to several advantages, which include rapid effect, minimal injury, and a short postoperative recovery period. With continuous increase in hyaluronic acid injections, many cases of hyaluronic acid injection-induced embolism have been reported. At present, methods for early treatment of hyaluronic acid injection–induced embolism include local injection of hyaluronidase, topical application of nitroglycerin ointment, massage, hot compression, and intravenous injections of antibiotics and hormones. Although early warm massage may facilitate hyaluronic acid degradation by hyaluronidase, local application of heat will also increase metabolic rate in the tissue, thereby reducing the ischemic tolerance of the tissue. Therefore, in this study, warm massage was limited to the first 30 minutes after hyaluronidase injection and was followed by local cooling using a gauze pad soaked with antibiotic saline solution. Excellent therapeutic effects were achieved with this approach. The methods of treatment for tissue ischemia caused by hyaluronic acid injection–induced embolism and clinical cases are introduced in the article.

Hyaluronic acid injection is 1 of most popular procedures used for facial rejuvenation and augmentation. It is widely used in the cosmetic surgery due to several advantages, which include rapid effect, minimal injury, and a short postoperative recovery period. With continuous increase in hyaluronic acid injections, many cases of hyaluronic acid injection–induced embolism have been reported. At present, methods for early treatment of hyaluronic acid injection–induced embolism include local injection of hyaluronidase, topical application of nitroglycerin ointment, warm massage, and intravenous injections of antibiotics and steroids.^1^ Although early local warm massage may facilitate hyaluronic acid degradation by hyaluronidase, local application of warm massage will also increase metabolic rate in the tissue, thereby reducing the ischemic tolerance of the tissue. Therefore, in this study, warm massage was limited to the first 30 minutes after hyaluronic acid injection, and was followed by local cooling using a gauze pad soaked with cold antibiotic saline solution. Excellent therapeutic effects were achieved with this approach, and are being reported as follows.

## CLINICAL DATA

From 2014 till date, a total of 9 patients were administered the antibiotic saline gauze local cooling as a step for treatment of tissue ischemic syndrome caused by hyaluronic acid injection–induced embolism (Table 1). All 9 patients were female, with age ranging from 20 to 35 years. Injection sites included the nasolabial folds (5 patients), alar groove (1 patient), glabellar lines (1 patient), and labial tubercle (1 patient), forehead augmentation (1 patient), and the injected volume of hyaluronic acid was 0.4–3 ml. The timing of postembolism hyaluronidase injection ranged between 5 minutes and 3 days postoperatively. Symptoms of patients noted during consultation included local persistent severe pain, while signs included dark purple patches and mottling of the skin in localized areas (**see figure, Supplemental Digital Content 1**, which displays clinical manifestations includes severe pain, dark purple patches, and mottling of the skin in localized areas in the early stage of tissue ischemia caused by hyaluronic acid injection-induced embolism. (a) Before treatment (b) After treatment, http://links.lww.com/PRSGO/A779). The most serious sign observed in patients was black scabs or blisters at the center of the lesion, with a history of scattered purulent spots for about 3 days.

**Table 1.**
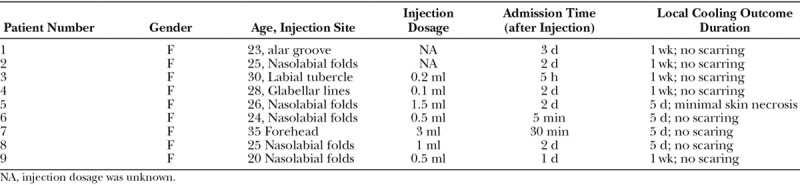
Patient Cases of Local Cooling Treatment of Tissue Ischemia Caused by Hyaluronic Acid Injection–induced Embolism

## TREATMENT METHODS

Each admitted patient was first injected with hyaluronidase at a concentration of 150–300 U/ml at sites filled with hyaluronic acid and areas showing abnormal skin color. The amount of injected hyaluronidase was used according to the introduction of consensus published in PRS.^1^ After injection, the affected sites were massaged for 30 minutes, followed by local cooling using an antibiotic saline-soaked gauze pad at each site. The method of local cooling was as follows: Antibiotic saline was refrigerated at 4°C for more than 2 hours. Then sterile gauze pads were soaked in the chilled antibiotic saline and applied at areas of skin showing dark purple discoloration. Gauze pads were replaced every 30 minutes, with a 30-minute rest period between 2 consecutive local coolings; alternatively, local cooling was reapplied only when the patient experienced localized pain. The patient was observed once per half an hour by nurse and twice a day by doctors. Local cooling is used at least 5 days for every patient who is suffered from HA-induced embolism in our unit. Systemic medication administered to patients included high doses of antibiotics and an intravenous infusion of dexamethasone at a dose of 5–10 mg/d over 3 consecutive days. Other treatment included hyperbaric oxygen therapy (4 patients) and topical nitroglycerin ointment (1 patient; Table 1).

## CASE REPORT

**Fig. 1.**
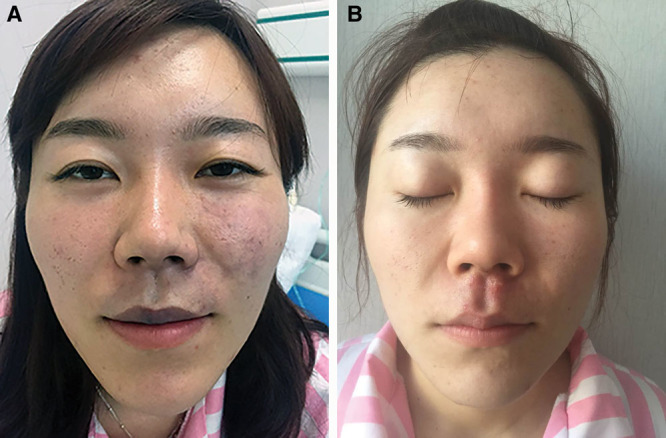
Embolism caused by the sharp needle injection of hyaluronic acid in the labial tubercle area. A, Dark purple discoloration of the upper lip and spotted contusions on the left side of the face were observed when the patient was admitted to the hospital (5 hours after the complication happened). B, Two weeks after treatment of the complication.

Patient 1, female, aged 30 years, received sharp needle injection in the labial tubercle area. After injection of about 0.2 ml of hyaluronic acid, the patient experienced sudden pain, and injection was stopped immediately. Even after 20 minutes, the pain did not subside; dark purple discoloration of the upper lip and spotted contusions on the left side of the face were observed (Fig. 1A). Two local injections of 1 ml of hyaluronidase at a concentration of 750 U/ml were administered 30 minutes apart, followed by continuous massage for 30 minutes. Five hours after injection, local cooling was applied using gauze pads soaked with saline containing gentamicin (80,000 units of gentamicin + 50 ml saline). Partial relief of pain was achieved after the local cooling. At the same time, hyperbaric oxygen therapy was administered once daily, and cefazolin sodium 2.0 g and 10 mg of dexamethasone were administered per day by intravenous infusion for 3 days. After treatment for up to 5 days postoperatively, 3 scattered purulent spots appeared at the base of the nasal columella; therefore, 0.5 ml of hyaluronidase at a concentration of 750 U/ml was injected again. One week after the injection, further disease progression was not observed; therefore, all treatment was discontinued. Two weeks after the injection, with exception of a residual 2 mm diameter scar at the junction of the left philtrum and the columella, as well as local pale purple ecchymosis, complete recovery was achieved at all other sites (Fig. 1B).

**Fig. 2.**
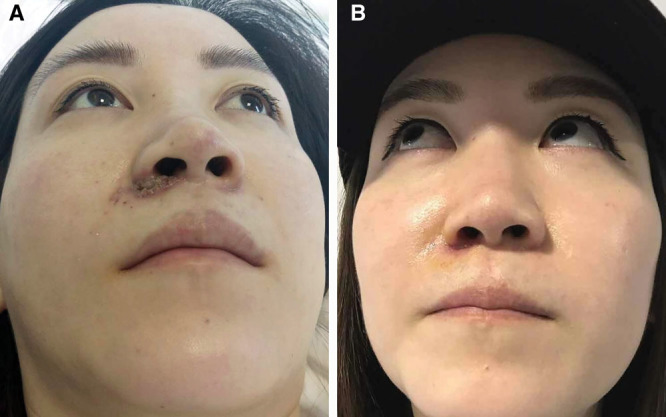
Complication of blunt needle injection of hyaluronic acid in the nasolabial folds. A, Skin ulcers and visible purulent spots appeared at the right alar base (3 days after the injection). B, After 10 days of treatment, the exposed skin was red in color without any obvious scarring.

Patient 2, female, aged 24 years, received blunt needle injection in the nasolabial folds. When the injection on the right side was almost completed, the patient experienced pain; however, this was not noticed by the injecting doctor. Six hours after the injection was completed, the injection site and adjacent area appeared bruised, and pain worsened. The patient developed localized oozing of blood. Two days after the injection, localized pain did not subside, and skin ulcers developed at the right alar base. Patient was admitted 3 days after injection, with skin ulcers at the right alar base and visible purulent spots. Immediately, injections of hyaluronidase at a concentration of 150–300 U/ml were administered at sites filled with hyaluronic acid and in areas showing abnormal skin color. After injection, the affected sites were massaged for 30 minutes, followed by local cooling using an antibiotic saline-soaked gauze pad applied to each site. Pain significantly reduced 2 hours after treatment. There was no increase in the area of skin ulceration and number of purulent spots after treatment. After continuous treatment for 5 days, pain disappeared, and scabs developed over the skin ulcers. After 10 days of treatment, the scabs fell off, and the exposed skin was red in color without any obvious scarring (Fig. 2).

## RESULTS

In all cases, local symptoms were considerably relieved within 3 days of treatment. In the patient who experienced the most severe side effects, significant pain relief and fading of skin discoloration were achieved 2 hours after treatment. There was no skin necrosis in 8 of the 9 cases. There was no significant increase in local purulent spots 5 days after injection in patients who were admitted 3 days after injection. One patient experienced significant local skin damage 3 days after treatment, and there was a small scar left, whereas 8 patients had intact skin with no scarring. All patients experienced residual redness in local skin areas after completion of treatment, which persisted for a maximum duration of 3 months.

## DISCUSSION

Conventional treatment of hyaluronic acid injection–induced embolism includes early local injection of hyaluronidase, topical application of nitroglycerin ointment, massage, warm massage, and intravenous injections of antibiotics and steroids. Based on the theory that an ice cap is used for brain protection in clinical treatment of cerebral ischemia, we believe that local cooling also helps to reduce the basal metabolic rate and increase the ischemic tolerance of tissues. Clinically, local cooling did reduce the early symptoms of localized pain and facilitated effective treatment of the 9 patients with hyaluronic acid injection–induced local embolism.

But, will local cooling causes vasoconstriction and aggravation of local ischemia?

Studies have shown that local cooling definitely results in local vasoconstriction.^2^ However, local cooling can also reduce the metabolic rate of local tissues. Moreover, at temperatures exceeding 25°C, local metabolic rate is reduced by 6–13% for every 1°C reduction in temperature^3^; however, under the same conditions, the change in free diffusion rate of blood is less than 1%.^4^ In addition, hyaluronic acid injection–induced embolism causes complete blockage from the site of embolization to the peripheral blood vessels. Under such conditions, though warm massage facilitates local vasodilation, it does not improve local circulation. Another study shows that local cooling can also reduce local reactive hyperemia.^5^ Thus, in the early treatment of hyaluronic acid injection–induced embolism, the benefits of local cooling outweigh its disadvantages.

In the future, the optimum temperature for local cooling is yet to be determined. Further studies are required to investigate the differences in treatment outcomes of cooling and warm massage in the treatment of skin ischemia caused by hyaluronic acid injection–induced embolism.
